# Body Listening and Resilience Among Elementary School Students: A Preliminary Study of the Roles of Social-Emotional Competence and Perceived Awareness

**DOI:** 10.3390/children13101353

**Published:** 2026-10-05

**Authors:** Hee Jung Min

**Affiliations:** The Department of Children and Adolescent Education, Dongguk University-WISE, Gyeongju 38066, Republic of Korea; hmin6@dongguk.ac.kr or aravindamhj@gmail.com

**Keywords:** body listening, interoceptive awareness, social-emotional competence, SEL, perceived awareness, attention, resilience, elementary school students

## Abstract

**Highlights:**

**What are the main findings?**

- Body listening was positively associated with resilience both directly and indirectly through social-emotional competence in elementary school students.
- Perceived awareness moderated the association between body listening and social-emotional competence, with the association becoming stronger as perceived awareness increased.

**What is the implication of the main finding?**

- The findings highlight social-emotional competence as a potential pathway linking attention to bodily signals with resilience in children.
- Both body listening and perceived awareness may be relevant to understanding children’s social-emotional functioning and resilience.

**Abstract:**

**Background:** This study examined the association between body listening and resilience among Korean elementary school students, focusing on the mediating role of social-emotional competence and the moderating role of perceived awareness. **Methods:** Participants were 78 students in Grades 3–6 from an elementary school in South Korea. Data were collected via surveys and analyzed using descriptive statistics, correlation analyses, mediation analysis, and moderated mediation analysis with the jAMM module in jamovi. **Results:** The results show that body listening was positively associated with both social-emotional competence and resilience. Social-emotional competence was a statistically significant partial mediator of the relationship between body listening and resilience. In addition, perceived awareness was a statistically significant moderator of the relationship between body listening and social-emotional competence, such that this association was stronger at higher levels of perceived awareness. These findings suggest that body listening is associated with resilience both directly and indirectly through social-emotional competence, and the association between body listening and social-emotional competence differed according to the level of perceived awareness. **Conclusion:** The findings indicate that fostering social-emotional competence and resilience among elementary school students requires helping them attend to bodily sensations and recognize their current physical and mental state.

## 1. Introduction

The rapid digital transformation of contemporary society and the widespread use of smartphones have prompted concerns about children’s psychological well-being. Haidt [1] found that the shift from play-based childhood to smartphone-based engagement has coincided with increases in anxiety, depression, and self-harm among children and adolescents. In the same vein, Brewer suggested that the immediate reward mechanisms of digital media can capture children’s attention repeatedly, reinforcing anxiety and emotional impulses [2]. Outcomes among Korean children have mirrored these broader patterns—emotional difficulties such as stress, depression, anxiety, and social isolation are increasingly common, and teachers have reported declines in students’ social-emotional competence and an increase in classroom conflict since the COVID-19 pandemic [3,4].

Against this backdrop, researchers and practitioners have paid increasing attention to social-emotional learning (SEL) as a way to support emotion regulation and the formation of constructive relationships among children, while identifying resilience as a quality that allows children to adapt to stress and adversity [5,6,7]. Because the elementary school years are a particularly important period for the development of self-awareness, emotion regulation, and interpersonal skills, research and interventions have focused on educational approaches that integrate SEL with mindfulness-related practices to support children’s emotional regulation, relationships, and resilience in everyday school settings [4,8]. SEL supports the development of competencies related to self-awareness, self-management, social awareness, relationship skills, and responsible decision-making [9,10]. In addition, this type of education is linked to children’s ability to regulate their emotions, maintain supportive relationships, and adapt to stress and adversity [4,11].

Recently, educators have extended SEL programs by incorporating mindfulness-related and body-based practices aimed at helping students attend to their internal experiences as part of self-awareness and self-regulation [6,8,12]. A central feature of mindfulness-related practice is learning to direct attention toward present-moment experience, including any sensations arising within the body. Paying attention to bodily experience is particularly relevant to social-emotional development because people often experience bodily changes before they fully recognize the corresponding emotions or express them in words. Children may notice muscle tension, changes in breathing, warmth, discomfort, or other bodily sensations before they clearly identify an emotional experience as anxiety, anger, or distress. Learning to attend to these bodily signals may help children recognize emotional changes earlier, allowing them to regulate their responses rather than reacting automatically. For this reason, mindfulness-informed, social-emotional, and trauma-informed educational approaches often teach children to notice bodily sensations as part of developing self-awareness and self-regulation [6,8,13,14].

This scholarly emphasis on bodily attention is closely related to research on interoception and interoceptive awareness. Interoception, which broadly refers to sensing and interpreting the signals that arise from within the body, is linked to emotional awareness, emotion regulation, social functioning, and resilience [15,16,17,18]. In this wider context, body listening refers to intentionally attending to bodily sensations and using them as information about emotional states and behavioral responses [18,19,20,21]. The practice of body listening can be understood as a mindfulness-related attentional process through which individuals turn toward bodily experience rather than ignoring or reacting automatically to it. For children, body listening may provide an embodied foundation for social-emotional competence by helping them use bodily cues to improve emotional understanding and identify adaptive responses.

Notably, however, simply attending to bodily sensations does not necessarily lead to emotion regulation or adaptive behavior. Rather, bodily signals only become meaningful for social-emotional functioning when an individual recognizes, interprets, and connects these signals with appropriate emotional and behavioral responses. In the present model, social-emotional competence was positioned as a mediator because it represents a broader set of emotional and interpersonal capacities than the relatively specific attentional orientation captured by body listening, whereas resilience reflects a broader capacity to adapt to stress and adversity. Thus, this ordering was theoretically specified to examine whether social-emotional competence statistically links body listening with resilience, rather than to establish a temporal or causal sequence. Specifically, noticing internal physical cues, understanding their emotional significance, regulating responses, and responding effectively in interpersonal situations may be associated with children’s ability to adapt to stress and adversity [6,8]. However, relatively little is known about how body listening is associated with social-emotional competence and, in turn, resilience.

Perceived awareness may further shape the relationship between body listening, social-emotional competence, and resilience. Notably, although awareness is closely related to attention, the two can be treated as analytically distinguishable processes—the latter involves directing and sustaining focus on one’s present experience, whereas the former entails clearly recognizing one’s current state [22,23,24]. In this sense, body listening reflects attention directed toward bodily signals, whereas perceived awareness reflects recognition of one’s current bodily and mental state. Importantly, attention is not a unitary process. Developmental research indicates that attention is composed of multiple, partly distinguishable components, including sustained, selective, and executive aspects of attention, which continue to develop across childhood. Recent research with primary school children further suggests that more specific attentional functions, such as focused, divided, and alternating attention, may show differential associations with learning and self-regulatory processes [25]. Within this framework, body listening as operationalized in the present study may be understood most closely as a self-reported tendency to allocate attention toward internal bodily signals rather than as a measure of general attentional ability or any specific neurocognitive component of attention. Thus, an individual can attend to bodily sensations without clearly recognizing those sensations. Theoretical accounts have linked both attention and awareness with self-regulatory functioning and adaptive responding [2].

Perceived awareness was positioned as a moderator because the present study specifically examined whether the association between attention to bodily signals and social-emotional competence varies according to children’s reported recognition of their current bodily and mental state. Thus, perceived awareness was treated not simply as an additional predictor, but as a factor that may be associated with the strength of the relationship between body listening and social-emotional competence. Specifically, the analysis assessed whether the association between body listening and social-emotional competence is stronger among children with higher perceived awareness, and social-emotional competence, in turn, is associated with greater resilience.

Although previous research has focused extensively on mindfulness and its relationships with depression, anxiety, stress, emotion regulation, and well-being in children [4,26,27,28,29], prior work has paid less attention to the specific role of body listening in social-emotional development. Specifically, few studies have examined how body listening, social-emotional competence, resilience, and perceived awareness operate within a single integrated model.

Accordingly, the present study examined the relationship between body listening and resilience among Korean elementary school students, focusing on the mediating role of social-emotional competence and the moderating role of perceived awareness. The analysis addressed the following research questions:

(1) Is there a significant association between body listening and resilience, and if so, does social-emotional competence mediate this relationship?
(2) Does perceived awareness moderate the relationship between body listening and social-emotional competence?

In sum, this study sought to clarify how attention to bodily signals and perceived awareness of one’s bodily and mental state are associated with social-emotional competence and resilience among elementary school students.

## 2. Materials and Methods

### 2.1. Participants

Participants were 78 students in Grades 3 through 6 attending an elementary school in a medium-sized city in South Korea. The school learned of the study through a research flyer and participated voluntarily. Data were collected after the Institutional Review Board of Dongguk University (DGU IRB 20250015) approved the study and the school granted permission. Before completing the survey, students were informed of the study purpose and procedures, anonymity and confidentiality measures, and their right to withdraw at any time. Consent was obtained from both students and their legal guardians. The questionnaire took approximately 30 min to complete. A total of 78 students completed the survey and were included in the final analysis.

Table 1 presents the demographic characteristics of the participants. The sample included 43 boys (55.1%) and 35 girls (44.9%). Of the students, 20 (25.6%) were in Grade 3, 32 (41.0%) were in Grade 4, 13 (16.7%) were in Grade 5, and 13 (16.7%) were in Grade 6.

### 2.2. Measures

#### 2.2.1. Body Listening

Body listening was assessed using the three-item Body Listening subscale of the Korean version of the Multidimensional Assessment of Interoceptive Awareness for Youth (MAIA-Youth), originally developed by Jones et al. [19] and adapted and validated in Korean by Kim [20]. The items were: “I listen for clues from my body about my emotions,” “When I am upset, I take time to check how my body feels,” and “I listen to my body to help me choose what to do.” Students responded on a 5-point Likert scale. In the present study, the overall scale showed excellent internal consistency (Cronbach’s α = 0.960), and the three-item Body Listening subscale showed good internal consistency (Cronbach’s α = 0.861). Inter-item correlations among the three Body Listening items ranged from 0.630 to 0.724 (all *p* < 0.001).

#### 2.2.2. Social-Emotional Competence

Social-emotional competence was assessed using the Social-Emotional Learning Scale developed by Coryn et al. [30], translated into Korean by Choi [31], and subsequently modified by Lee [32] for elementary school students. The scale consists of 20 items across three domains: task articulation (6 items), peer relationships (7 items), and self-regulation (7 items). Students responded on a 5-point Likert scale. Cronbach’s α for the total scale was 0.953 in the present study.

#### 2.2.3. Resilience

Resilience was assessed using a 28-item scale for elementary school students adapted by Kang [5], based on the original scale developed by Reivich and Shatte [33]. The scale includes seven domains: emotion regulation, impulse control, realistic optimism, causal analysis, empathy, self-efficacy, and reaching out, with four items in each domain. All items were rated on a 5-point Likert scale. Cronbach’s α for the total scale was 0.967 in the present study.

#### 2.2.4. Perceived Awareness

Perceived awareness was assessed using a single researcher-developed item: “I am aware of the state of my body and mind.” The item was designed as a brief indicator of children’s perceived awareness of their current bodily and mental state. The item was rated on a 5-point Likert scale, with higher scores indicating greater perceived awareness.

### 2.3. Data Analysis

Data were analyzed using the jAMM (jamovi Advanced Mediation Models) module in jamovi version 2.4 [34,35]. First, descriptive statistics were calculated to examine the distributions of the major variables, and Pearson’s correlations were used to examine the associations among them. Variance Inflation Factors (VIFs) were also calculated to assess multicollinearity. In the next step, mediation analysis was conducted to examine whether social-emotional competence mediated the association between body listening and resilience. A moderated mediation analysis was then performed within the meditation model to determine whether perceived awareness moderates the indirect association between body listening and resilience through social-emotional competence. Interaction effects were examined for the association between body listening and social-emotional competence, the association between social-emotional competence and resilience, and the direct association between body listening and resilience. All continuous predictors involved in interaction terms were mean-centered before analysis [36]. Indirect and conditional indirect effects were evaluated using 5000 bootstrap samples and 95% confidence intervals. Conditional indirect effects were estimated at low (−1 SD), mean, and high (+1 SD) levels of perceived awareness. Given the relatively small sample, the moderation analysis was interpreted as preliminary, with particular attention to the magnitude and uncertainty of the interaction effect. 

## 3. Results

### 3.1. Descriptive Statistics and Correlations

Descriptive statistics by gender and grade are presented in Table 2, Panel A. Grade-level descriptive statistics showed some variation across the four study variables. Grade 3 students had the highest mean scores and Grade 5 students the lowest mean scores for body listening, social-emotional competence, resilience, and perceived awareness, with mean scores increasing again in Grade 6. Thus, the descriptive pattern did not show a consistent increase or decrease across grade levels.

Overall descriptive statistics and zero-order Pearson’s correlations among body listening, social-emotional competence, resilience, and perceived awareness are presented in Table 2, Panel B. All variables were positively and significantly correlated (*p* < 0.001). Resilience was strongly correlated with social-emotional competence (*r* = 0.863) and body listening (*r* = 0.788). Body listening was also positively correlated with social-emotional competence (*r* = 0.638) and perceived awareness (*r* = 0.532).

### 3.2. Multicollinearity Diagnostics

Multicollinearity was examined before conducting mediation analyses. VIF values ranged from 1.42 to 1.88, and tolerance values ranged from 0.531 to 0.706, indicating no evidence of problematic multicollinearity among the predictors.

### 3.3. Mediating Role of Social-Emotional Competence

A mediation analysis was conducted to examine whether social-emotional competence mediated the association between body listening and resilience. As shown in Table 3, body listening was positively associated with social-emotional competence (B = 2.820, *SE* = 0.385, β = 0.638, *p* < 0.001), which, in turn, was positively associated with resilience (B = 0.890, *SE* = 0.086, β = 0.607, *p* < 0.001). The direct association between body listening and resilience also remained significant (B = 2.592, *SE* = 0.380, β = 0.401, *p* < 0.001). The indirect association between body listening and resilience through social-emotional competence was statistically significant (B = 2.509, *SE* = 0.420, β = 0.388, *p* < 0.001), with a 95% bootstrap confidence interval of [1.551, 3.90]. Because both the indirect and direct associations were statistically significant, the results were consistent with partial mediation. The total association between body listening and resilience was also significant (B = 5.101, *SE* = 0.454, β = 0.788, *p* < 0.001).

### 3.4. Moderation Analysis Within the Meditation Model

A moderated mediation analysis was conducted to examine whether the indirect association between body listening and resilience through social-emotional competence varied as a function of perceived awareness. As shown in Table 4, the interaction between body listening and perceived awareness was significantly associated with social-emotional competence (B = 0.935, *SE* = 0.467, β = 0.17, *p* = 0.04). Thus, the association between body listening and social-emotional competence varied according to the level of perceived awareness. The interaction between body listening and perceived awareness in relation to resilience was not statistically significant (B = 0.493, *SE* = 0.517, β = 0.06, *p* = 0.34). Similarly, the interaction between social-emotional competence and perceived awareness in relation to resilience was not statistically significant (B = −0.233, *SE* = 0.129, β = −0.59, *p* = 0.07). Thus, a significant moderation was observed specifically in the association between body listening and social-emotional competence. The standardized interaction coefficient for this association (β = 0.17) indicated a modest moderation effect.

### 3.5. Conditional Indirect Effects 

Conditional indirect effects were estimated at low (−1 SD), mean, and high (+1 SD) levels of perceived awareness. As shown in Table 5, the indirect association between body listening and resilience through social-emotional competence was statistically significant at all three levels of perceived awareness. The estimated indirect effect was 1.703 at low perceived awareness (*p* = 0.006), 2.037 at the mean level (*p* < 0.001), and 2.082 at high perceived awareness (*p* < 0.001). Although the conditional indirect association increased across levels of perceived awareness, the differences in magnitude were modest. Because these conditional effects were estimated from the moderated mediation model, they are not identical to the indirect effect estimated from the simple mediation model presented in Table 3.

## 4. Discussion

The present study examined the association between body listening and resilience among elementary school students, focusing on the mediating role of social-emotional competence and the moderating role of perceived awareness. Body listening was positively associated with resilience both directly and indirectly through social-emotional competence. In addition, perceived awareness modestly moderated the association between body listening and social-emotional competence. Taken together, these findings suggest that attention to bodily signals and awareness of one’s present bodily and mental state may play related but complementary roles in children’s social-emotional development and adaptation.

First, social-emotional competence significantly mediated the association between body listening and resilience. Children who reported greater body listening also tended to report higher social-emotional competence, which, in turn, was associated with greater resilience. This finding is consistent with the view that bodily signals provide information salient to emotional recognition and regulation. Children may experience changes in breathing, tension, body temperature, or discomfort before they can clearly identify or verbalize their emotions. Attending to such signals may be associated with greater recognition of emotional changes, bodily experience, self-regulation, and adaptive interpersonal responses [4,6].

The partial nature of the mediation further shows that social-emotional competence represents an important, but not exclusive, pathway linking body listening with resilience. Given that body listening remained directly associated with resilience after social-emotional competence was included in the model, attention to bodily signals may also be associated with other regulatory processes related to resilience, such as earlier recognition of stress responses, regulation of physiological arousal, or greater flexibility in responding to internal experience [13,14,17]. Thus, body listening may be associated with resilience through both social-emotional functioning and other processes related to the recognition and regulation of internal experience.

At the same time, the strong correlation between social-emotional competence and resilience suggests some conceptual overlap between the two constructs, particularly in domains related to self-regulation and interpersonal functioning. Accordingly, the indirect association should be interpreted cautiously as reflecting related, but not necessarily fully distinct, psychological processes.

Second, perceived awareness moderated the association between body listening and social-emotional competence such that the association became stronger as perceived awareness increased. However, because perceived awareness was assessed using a single researcher-developed item, this finding should be interpreted cautiously. The result should be understood as pertaining specifically to children’s perceived recognition of their current bodily and mental state rather than to awareness as a broader psychological construct. This observed pattern is consistent with an analytical distinction between body listening as a mindfulness-related attentional process and perceived awareness as the recognition of one’s current bodily and mental state. For example, while mindfulness includes purposefully paying attention to present-moment experience [37], theoretical accounts have distinguished between attentional engagement and broader processes of monitoring and recognizing ongoing experience [22,23,24]. This interpretation is also consistent with recent meta-analytic evidence showing that mindfulness-based interventions were associated with improvements in self-reported interoceptive awareness and that improvements in interoception were related to reduction in psychological distress [38]. However, the present findings indicate that the association between body listening and social-emotional competence differs according to children’s reported level of perceived awareness and do not establish a causal or directional relationship among these variables.

In the present model, body listening reflects attention directed toward bodily sensations, whereas perceived awareness entails children’s recognition of their present bodily and mental state. Although these processes are closely related, they may serve complementary rather than identical functions. Attention to bodily sensations may provide access to internal signals, whereas perceived awareness may reflect the extent to which those internal states are consciously recognized. This interpretation is broadly consistent with Brewer’s view that attention and awareness are closely related to self-regulatory processes and adaptive responding [2].

The results for conditional indirect effects provide further context for this interpretation. The indirect association between body listening and resilience through social-emotional competence was statistically significant at low, mean, and high levels of perceived awareness. Although the estimated indirect association increased as perceived awareness increased, the differences were modest. Thus, the indirect association was evident across levels of perceived awareness, while the association between body listening and social-emotional competence was somewhat stronger at higher levels of perceived awareness. Attending to bodily sensations may therefore be particularly relevant to children’s social-emotional functioning when they are also able to recognize and understand their present bodily and mental state.

These findings have several implications for social-emotional education. Contemporary SEL approaches increasingly incorporate mindfulness-related and body-based practices that guide students to attend to present-moment experience and bodily sensations as part of developing self-awareness and self-regulation [6,8,12,38]. The present findings suggest the potential value of integrating two complementary processes into such approaches: directing attention toward bodily experience and recognizing the meaning of one’s present internal state.

In practice, children may first be guided to notice changes in breathing, tension, warmth, discomfort, or other bodily sensations and then be supported in recognizing how these sensations relate to emotions and possible responses. Such practices may be relevant to the connection between bodily experience, emotional understanding, and adaptive responses. In this sense, educators may cultivate mindfulness-related bodily attention and awareness jointly in social-emotional education. The educational significance of this approach lies in helping children not only direct attention toward internal experience but also recognize, understand, and use bodily information in emotionally and socially meaningful ways.

The findings also point toward a broader understanding of social-emotional competence as an embodied process. Social-emotional development is often described in terms of identifying emotions, regulating behavior, and managing relationships. The present findings indicate that these capacities may be associated with how children attend to and interpret their bodily experience. From this perspective, bodily signals provide an important source of information for recognizing emotional changes and responding to them with greater flexibility. Thus, integrating a joint focus on bodily attention and emotional understanding may be a meaningful direction for the continued development of social-emotional and resilience education.

Several limitations of the analysis should be acknowledged. First, the study relied entirely on self-report measures, and perceived awareness was assessed using a single researcher-developed item, which did not allow estimation of internal consistency and for which independent psychometric validation was not available. Further, the study did not use parallel validated measures to assess mindfulness-related attention and awareness. Therefore, the distinction proposed here between bodily attention and perceived awareness should be understood as theoretically informed and preliminary rather than as a definitive empirical separation of the two constructs. Future research should use validated, developmentally appropriate multi-item measures; examine discriminant validity more directly; and incorporate multiple sources of information, including teacher reports, behavioral measures, interviews, and observations.

Second, the study included only 78 students from a single elementary school, limiting the generalizability and stability of the findings. This limitation is particularly relevant given the complexity of the moderated mediation model and the modest magnitude of the interaction effect. Although the interaction between body listening and perceived awareness was statistically significant, its magnitude was modest and should therefore be regarded as preliminary. Replication with larger and more diverse samples is needed to examine the robustness of the observed associations.

Third, the cross-sectional study design cannot establish temporal order or causal relationships. The mediation and moderation findings should therefore be understood as statistical associations consistent with the proposed theoretical model rather than evidence of developmental or causal pathways. Reverse or reciprocal relationships are plausible. In addition, the study did not evaluate a specific intervention. Longitudinal and intervention studies are needed to clarify the direction of these relationships.

## 5. Conclusions

The present study examined the association between body listening and resilience among elementary school students, focusing on the mediating role of social-emotional competence and the moderating role of perceived awareness. Body listening was related to resilience both directly and indirectly through social-emotional competence. Perceived awareness also moderated the association between body listening and social-emotional competence, although the magnitude of the interaction was modest. The findings provide preliminary evidence that attention to bodily sensations and awareness of one’s present bodily and mental state are related in complementary ways to children’s social-emotional development. Longitudinal and intervention studies are needed to clarify the direction of these associations and their educational significance.

## Figures and Tables

**Table 1:** Demographic Characteristics of Participants (*N* = 78).

	Group	Frequency (*N*)	Percentage (%)
Gender	Male	43	55.1
	Female	35	44.9
Grade	3rd	20	25.6
	4th	32	41.0
	5th	13	16.7
	6th	13	16.7
Total		78	100

**Table 2:** Descriptive Statistics and Correlations Among the Major Variables (*N* = 78).

Panel A. Descriptive Statistics by Gender and Grade						
Variable	Boys (*n* = 43) M (SD)	Girls (*n* = 35) M (SD)	Grade 3 (*n* = 20) M (SD)	Grade 4 (*n* = 32) M (SD)	Grade 5 (*n* = 13) M (SD)	Grade 6 (*n*= 13) M (SD)
Body Listening	13.0 (3.68)	13.3 (4.03)	14.4 (3.69)	13.3 (4.29)	10.9 (2.96)	12.8 (2.68)
Social-Emotional Competence	76.3 (17.4)	81.4 (15.9)	84.8 (12.7)	77.2 (18.8)	68.9 (18.5)	81.8 (11.3)
Resilience	102 (25.8)	111 (22.6)	116 (21.7)	105 (28.2)	98.1 (18.3)	101 (22.6)
Perceived Awareness	3.91 (0.81)	4.14 (0.81)	4.25 (0.91)	4.06 (0.80)	3.62 (0.77)	3.92 (0.64)
Panel B. Overall Descriptive Statistics and Correlations						
Variable	1	2	3	4	Mean	SD
Body Listening	—				13.1	3.82
Social-Emotional Competence	0.638 ***	—			78.6	16.9
Resilience	0.788 ***	0.863 ***	—		106	24.7
Perceived Awareness	0.532 ***	0.563 ***	0.674 ***	—	4.0	0.81

*** *p* < 0.001.

**Table 3:** Mediation Analysis of the Association Between Body Listening and Resilience Through Social-Emotional Competence.

Effect	Path	B	*SE*	β	*z*	*p*	95% CI
Indirect Effect	Body Listening → Social-Emotional Competence → Resilience	2.509	0.420	0.388	5.97	<0.001	[1.551, 3.90]
Direct Effect	Body Listening → Resilience	2.592	0.380	0.401	6.81	<0.001	[1.517, 3.57]
Total Effect	Body Listening → Resilience	5.101	0.454	0.788	11.24	<0.001	[4.209, 5.96]
Path A	Body Listening → Social-Emotional Competence	2.820	0.385	0.638	7.32	<0.001	[2.05, 3.61]
Path B Resilience	Social-Emotional Competence →	0.890	0.086	0.607	10.32	<0.001	[0.647, 1.24]

Note: Confidence intervals computed with bootstrap percentiles. Betas are completely standardized effect sizes.

**Table 4:** Moderating Effects of Perceived Awareness.

Path	B	*SE*	β	*p*
Body Listening * Awareness → Social-Emotional Competence	0.935	0.467	0.17 *	0.04
Body Listening * Awareness → Resilience	0.493	0.517	0.06	0.34
Awareness * Social-Emotional Competence → Resilience	−0.233	0.129	−0.59	0.07

* *p* < 0.05.

**Table 5:** Conditional Indirect Effects by Level of Perceived Awareness.

Awareness	Indirect Effect	*SE*	β	*z*	*p*	95% CI
Low(−1SD)	1.703	0.615	0.252	2.77	0.006	[0.398, 3.55]
Mean	2.037	0.420	0.316	4.85	<0.001	[0.936, 3.63]
High(+1SD)	2.082	0.465	0.334	4.48	<0.001	[0.603, 4.25]

## Data Availability

The data that support the findings of this study are available from the author upon reasonable request.
